# Mechanism of ameliorating cerebral ischemia/reperfusion injury by antioxidant inhibition of autophagy based on network pharmacology and experimental verification

**DOI:** 10.18632/aging.205773

**Published:** 2024-04-25

**Authors:** Yuantie Zheng, Zhicheng Huang, Yang Zhao, Lili Huang, Jun Wang, Heping Li, Xing Chen, Jingsong Wang, Jingwen Xie

**Affiliations:** 1 Department of Pharmacy, The Second Affiliated Hospital and Yuying Children’s Hospital of the Wenzhou Medical University, Wenzhou, Zhejiang, China; 2 Department of Pharmacy, Ezhou Central Hospital, Ezhou, Hubei, China; 3 Department of Pharmacy, Lihuili Hospital Affiliated to Ningbo University, Ningbo, Zhejiang, China; 4 Department of Health, Chongqing Industry and Trade Polytechnic, Chongqing, China; 5 Department of Pharmacy, Guangyuan Central Hospital, Guangyuan, Sichuan, China

**Keywords:** network pharmacology, stroke, autophagy, SIRT1

## Abstract

Cerebral ischemia-reperfusion injury (CIRI) is one of the most difficult challenges in cerebrovascular disease research. It is primarily caused by excessive autophagy induced by oxidative stress. Previously, a novel compound X5 was found, and the excellent antioxidant activity of it was verified in this study. Moreover, network pharmacological analysis suggested that compound X5 was closely associated with autophagy and the mTOR pathway. *In vitro*, X5 could significantly inhibit the expression of autophagy proteins Beclin-1 and LC3-β, which are induced by H_2_O_2_, and promote the expression of SIRT1. *In vivo*, compound X5 significantly reduced the infarct size and improved the neurological function scores in the middle cerebral artery occlusion (MCAO) model of rats. In conclusion, ROS-induced autophagy is closely related to mTOR, SIRT1 and others, and X5 holds promise as a candidate for the treatment of CIRI.

## INTRODUCTION

Stroke, a common cerebrovascular disease, causes significant pressure on families and communities due to its high mortality and disability. Approximately 80% of all stroke cases are attributed to ischemic stroke [1, 2]. Intravenous thrombolysis with recombinant tissue plasminogen activator (rtPA) is the most effective treatment for ischemic stroke; however, owing to its narrow therapeutic window, the high mortality and disability rate remain a challenge [3]. This may primarily be attributed to the reperfusion of blood after thrombolysis leading to a series of deleterious secondary events, known as cerebral ischemia-reperfusion injury (CIRI) [4, 5].

As one of the core mechanisms of CIRI, oxidative stress induced by excessive reactive oxygen species (ROS) has been widely discussed [6, 7]. This is because excessive ROS can cause cell dysfunction and death through the oxidation of protein, DNA, and RNA, which can lead to brain tissue damage [8]. Notably, excessive autophagy induced by oxidative stress has recently been shown to play a critical role in brain injury [9, 10]. Across diverse eukaryotic organisms, autophagy serves as a cellular defense mechanism, characterized by “self-phagocytosis, and plays a key role in the fundamental renewal of cellular components and responds actively to organelle injury and nutritional deprivation [11–14]. However, excessive autophagy induced by oxidative stress can induce apoptosis and aggravate brain injury [10, 15, 16]. Therefore, reducing oxidative stress-induced autophagy may provide novel therapeutic strategies for the prevention and treatment of CIRI.

**Figure 1:**
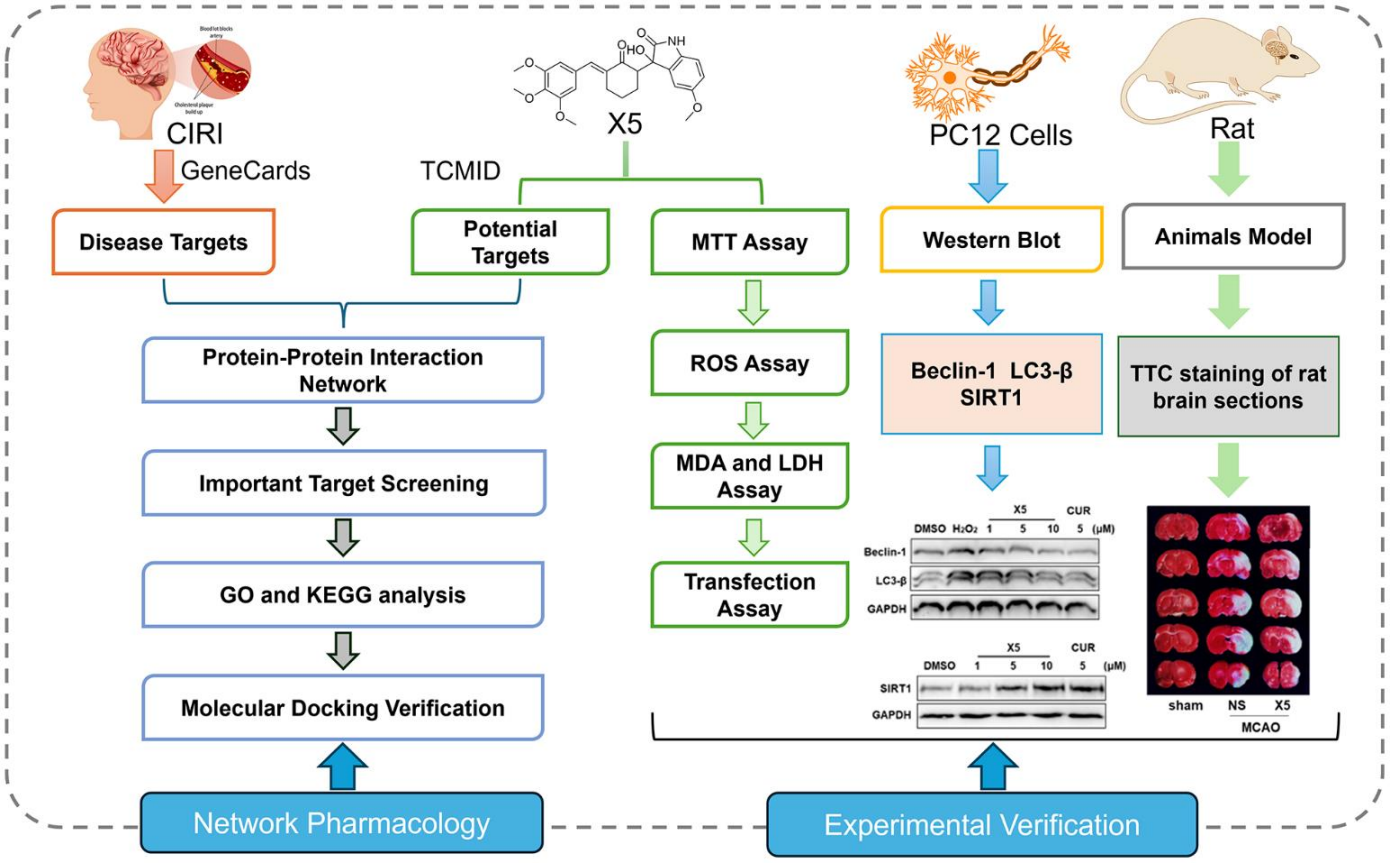
Comprehensive technical pathway integrating network pharmacology and experimental verification.

Network pharmacology, initially proposed by Hopkins, is a method that combines high-throughput data integration, database retrieval, data mining, target prediction, and *in silico* experiments [17]. In theory, it enables the analysis of the mode of action of small molecules at a network level, allowing for the analysis and prediction of their mechanisms, efficacy, and toxicity. Moreover, it facilitates the design of novel drugs with improved therapeutic effects and clinical safety [18, 19]. We designed a new antioxidant compound X5 to investigate the mechanism of oxidative stress-induced autophagy. Subsequently, we explored the relationship between the antioxidant compound X5 and autophagy by a combination of network pharmacology and experimental validation. Using network pharmacology, we identified an interaction between X5 and autophagy-related proteins. Subsequently, we assessed the effect of X5 on oxidative stress-induced autophagy in PC12 cells *in vitro*. Furthermore, we also assessed the protective effects of X5 in rats after MCAO. In conclusion, this article explored the relationship between the antioxidant compound X5 and autophagy using a combination of network pharmacology and experimental validation, and X5 may be a promising compound for the treatment of CIRI (Figure 1).

## RESULTS

### Antioxidant compound X5 alleviated oxidative stress in PC12 cells

**Figure 2:**
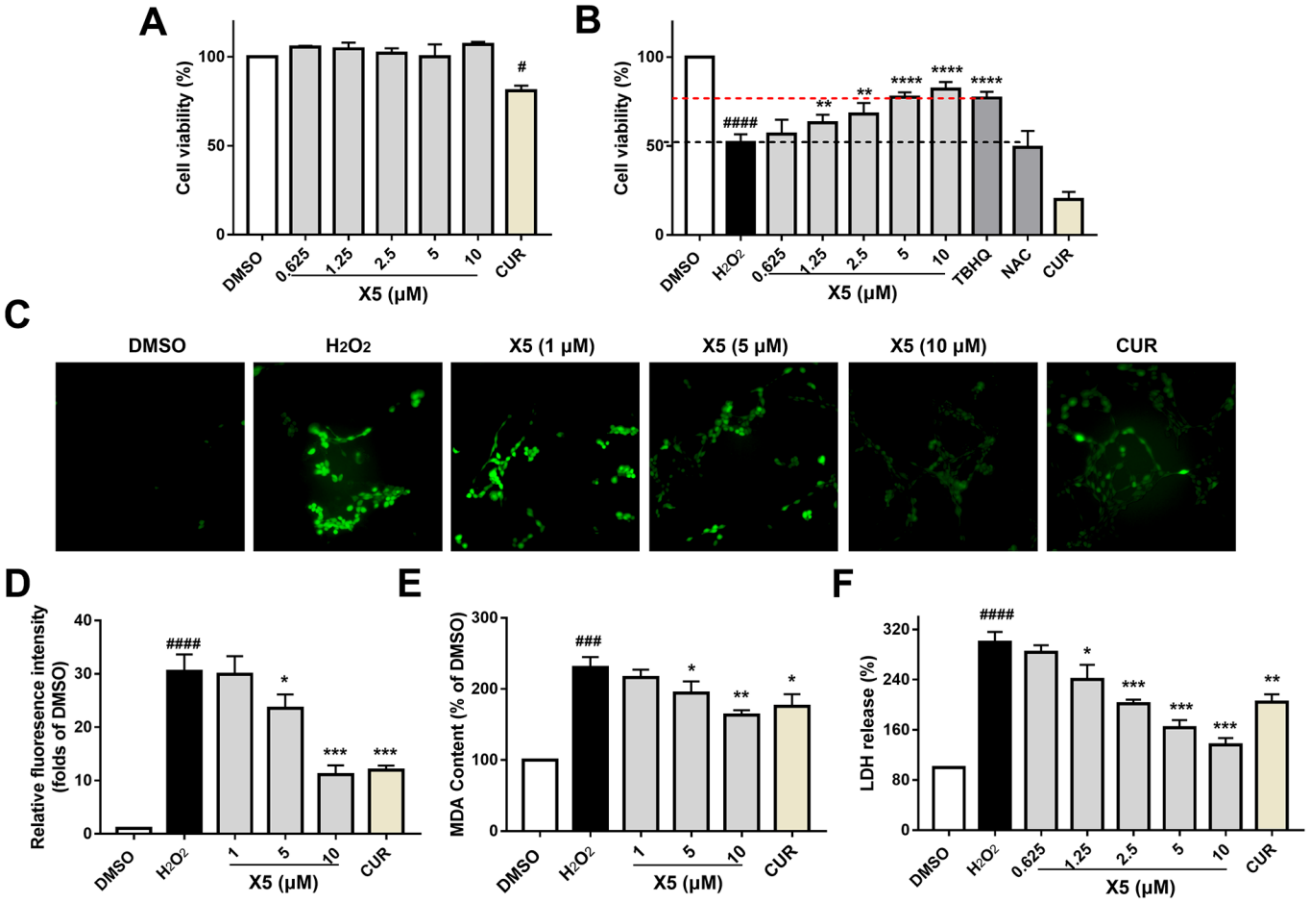
**X5 reduced H_2_O_2_-induced oxidative stress injury in PC12 cells.** PC12 cells were incubated with X5 (0.625, 1.25, 2.5, 5 and 10 μM), CUR, TBHQ and NAC (10 μM) for 42 h (**A**) and 18 h (**B**), cells were incubated with H_2_O_2_ (600 μM) for 24 h (**B**). Cell viability was measured by MTT assay. X5 (1, 5 and 10 μM) at different concentrations and CUR (5 μM) were pre-incubated on PC12 cells for 18 h, followed by stimulation with H_2_O_2_ (800 μM) for 3 h (**C**, **D**) or 6 h (**E**), and ROS and MDA levels were measured according to the manufacturer’s methods. (**F**) X5 inhibited LDH release in a dose dependent manner. PC12 cells were exposed to different concentrations of X5 and CUR (5 μM) for 18 h, then the cells were treated with H_2_O_2_ (600 μM) for 24 h, and LDH release was detected according to the manufacturer’s methods. The data are expressed as mean ± SD, n ≥3. ^####^p<0.0001, ^###^p<0.0001, ^#^p<0.05 vs DMSO, ^****^p<0.0001, ^***^p<0.001, ^**^p<0.01, ^*^p<0.05 vs H_2_O_2_.

In previous studies, we identified a novel compound X5 with excellent antioxidant activity, but its effectiveness on nerve cells remains to be investigated. PC12 cells are derived from a rat pheochromocytoma; they exhibit neuronal-like characteristics and can differentiate into cell types resembling neurons. In this study, PC12 cells were incubated with different concentrations of X5 and curcumin (CUR) (5 μM) for 42 h, and their toxicity to PC12 cells was assessed (Figure 2A). X5 showed almost no toxic effects on PC12 cells. The H_2_O_2_-induced damage model is a common oxidative stress injury model *in vitro*. PC12 cells were incubated with X5, curcumin, TBHQ, and NAC for 18 h, followed by H_2_O_2_ treatment for 24 h. Subsequently, their antioxidant activity was assessed by a MTT assay, as shown in Figure 2B. The viability of PC12 cells after H_2_O_2_ damaged was approximately 51%; however, pre-incubation with X5 and TBHQ maintained cell viability to over 80%, whereas curcumin and NAC conferred no protection to cells.

PC12 cells treatment with H_2_O_2_ increased ROS and MDA levels, toxicity of cells, and promoted the LDH release. However, PC12 cells pre-incubated with X5 significantly prevented the increase in ROS and MDA levels (Figure 2C–2E), as well as inhibited the release of LDH after stimulation of H_2_O_2_ in a concentration-dependent manner (Figure 2F). Therefore, X5 protected PC12 cells from H_2_O_2_-induced oxidative stress damage by decreasing the levels of ROS, MDA, and LDH.

### Construction of a “compound – protein” network

**Figure 3:**
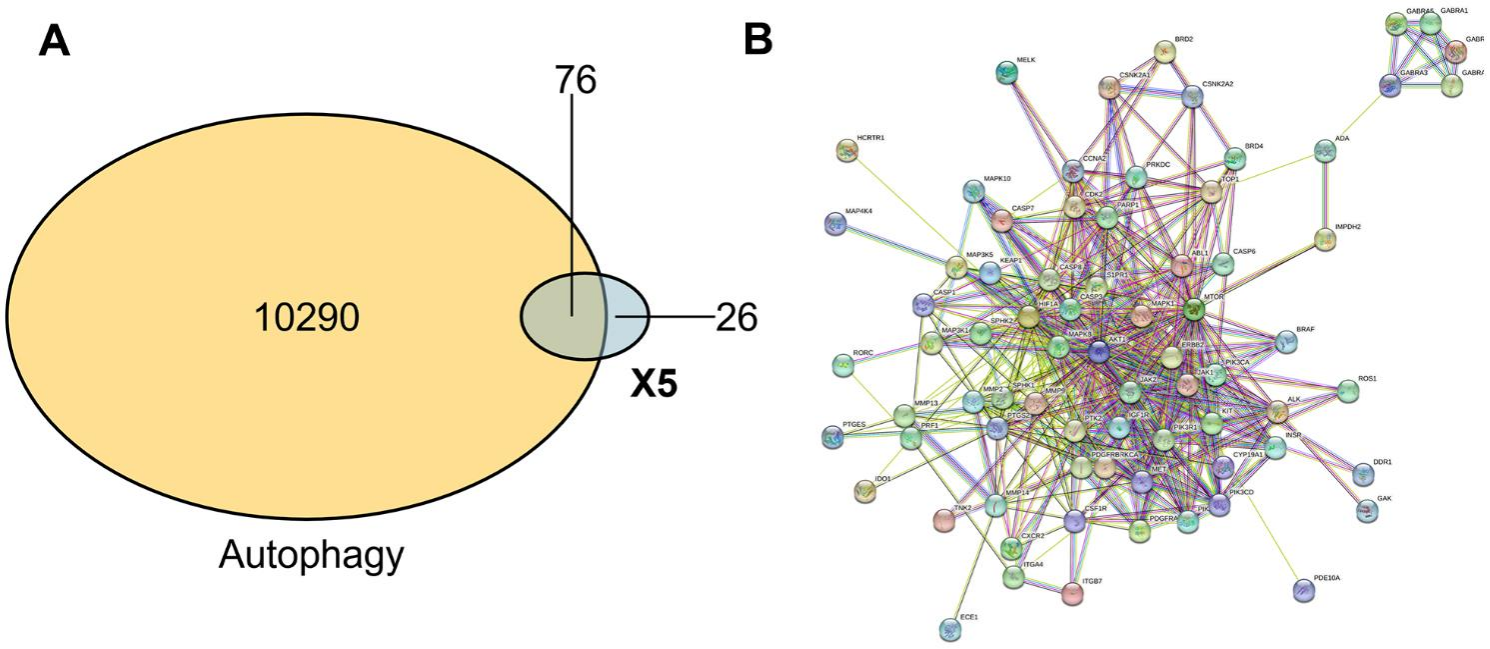
**The compound-protein network.** (**A**) Common target of small molecule drug X5 and autophagy. (**B**) Interaction network between small molecule drug X5 and autophagy.

To further study the mechanism between X5 and autophagy, we substituted X5 to network pharmacology. We imported the structure of compound X5 into the SwissTargetPrediction database for prediction, identified its potential targets, and finally obtained 92 effective targets after calibration and weight removal using the UniProt database. A total of 10,290 targets related to autophagy were identified using the Disgenet database. Subsequently, through the combination tool VennDetail Shiny, as shown in Figure 3A, we identified 76 common targets between X5 and autophagy, indicating that X5 has a great impact on autophagy-related pathways. The 76 common targets were imported into the STRING database, the confidence interval was adjusted to 0.4, the disconnected nodes were hidden, and the corresponding protein interaction network (PPI) was finally obtained (Figure 3B). The nodes represent the target of action, and the lines represent the interaction between the targets. The line thickness indicated high support for the data and high confidence.

### Discovery of hub protein

**Figure 4:**
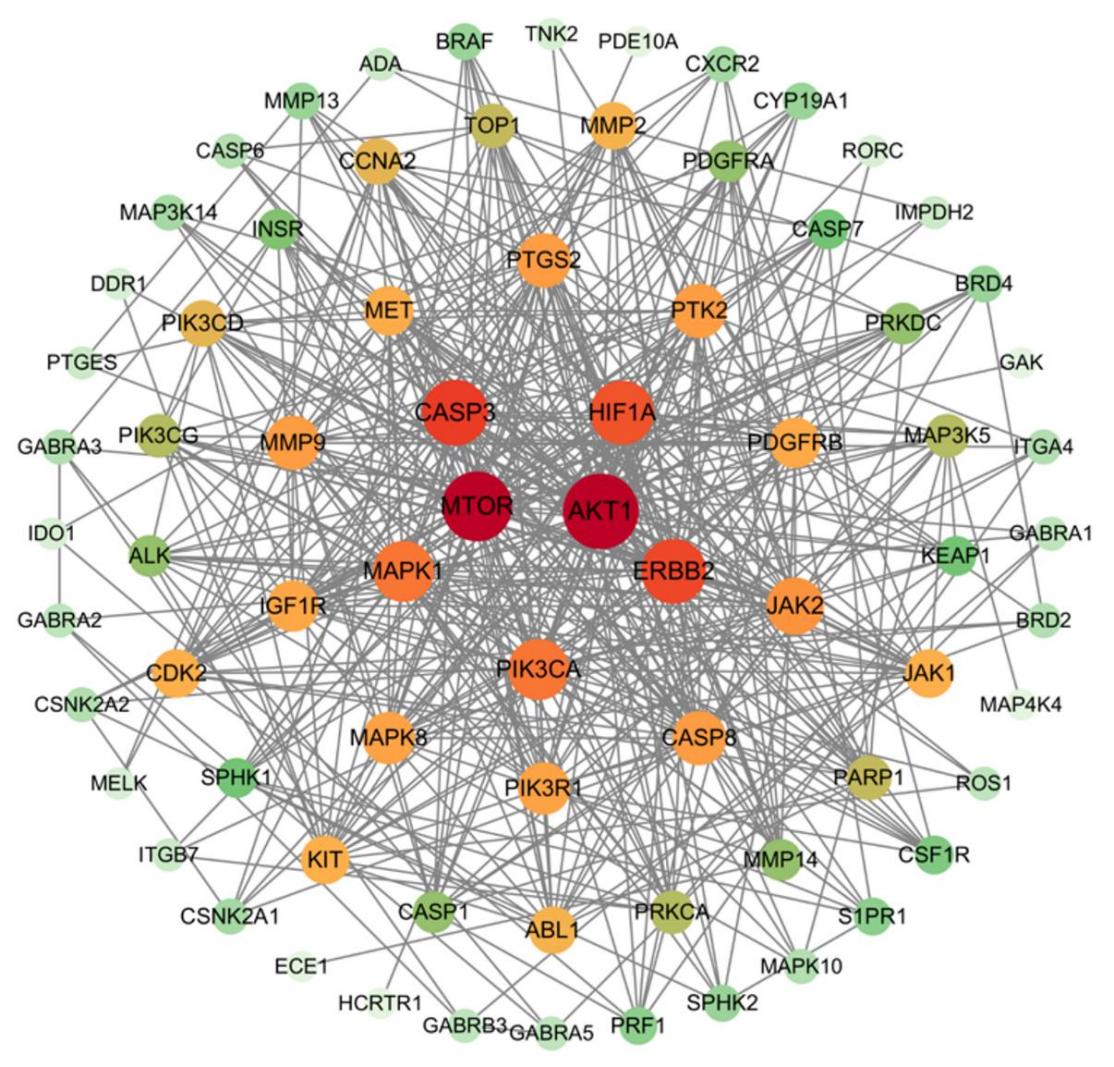
Key target of small molecule drug X5 and autophagy.

The network diagram obtained from STRING was subjected to Cytoscape software for PPI network topology attribute analysis. The average degree of the network was 14.83 and the average betweenness was 3.74c×10^-2^. Nodes with both degree and betweenness greater than the average were selected as the key targets. Results Visual analysis was conducted as shown in Figure 4. The size and color intensity of nodes directly correlate with the degree value of the target, indicating that larger characters and more vibrant colors represent higher degrees. Hub proteins mTOR, AKT1, CASP3, HIF1A and ERBB2 were finally identified.

### The mechanism of X5 inhibiting autophagy

**Figure 5:**
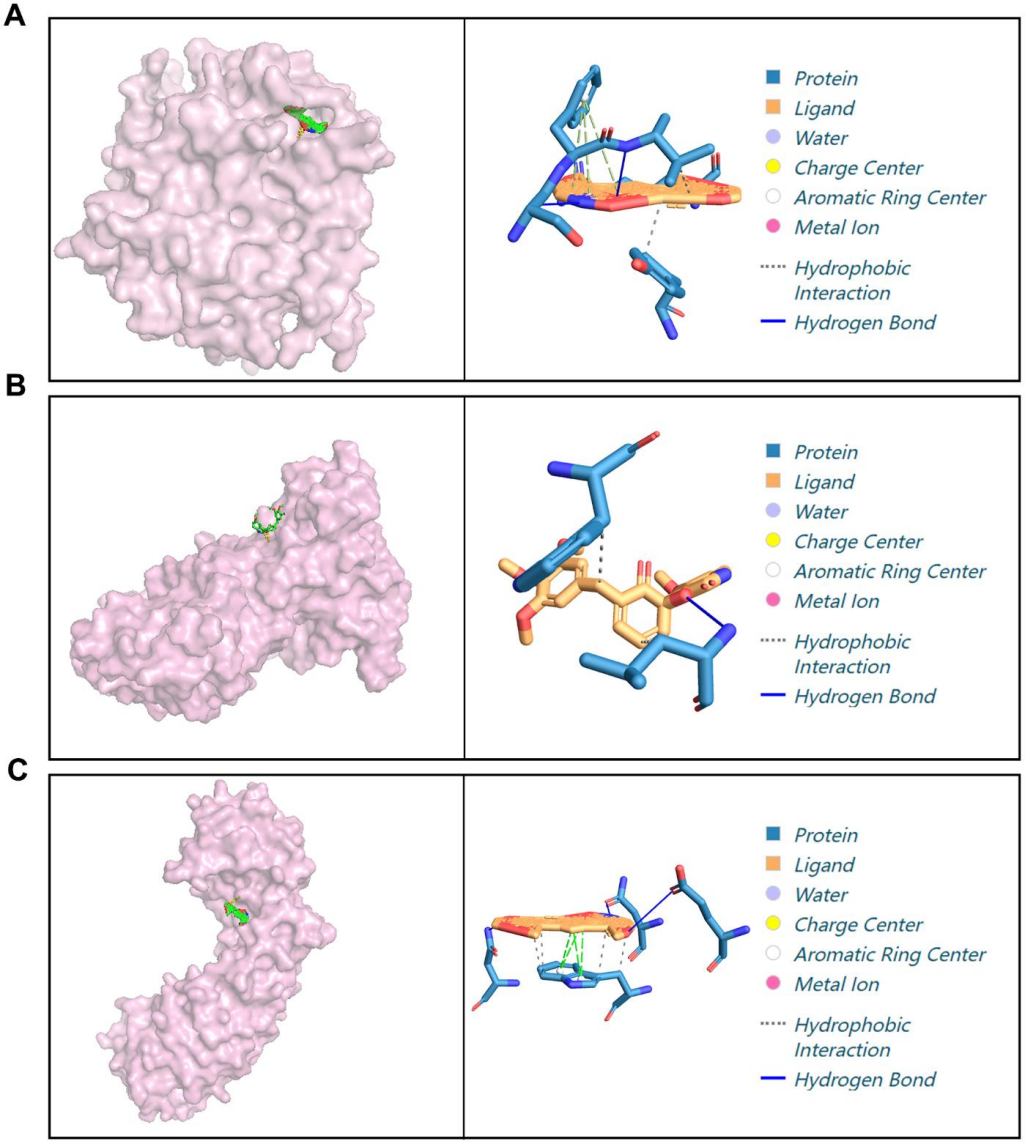
**Docking of X5 with different proteins.** (**A**) Molecular docking of X5 with CASP3. (**B**) Molecular docking of X5 with ERRB2. (**C**) Molecular docking of X5 with HIF1A.

To study the mechanisms underlying the autophagy inhibition activities of X5, molecular docking experiments were performed between compounds X5 and mTOR, AKT1, CASP3, HIF1A, ERBB2 (Figure 5). Compound X5 occupied the kinase domain of CASP3, ERRB2, HIF1A with low binding energies of -16.0, -14.8, -13.0 kcal/mol, respectively, indicating highly stable binding. However, X5 showed poor binding affinity with mTOR and AKT1, requiring additional energy for binding. Therefore, we speculate that X5 inhibits autophagy by binding CASP3, ERRB2, or HIF1A, subsequently suppressing the expression of mTOR and AKT1.

### GO and KEGG enrichment analysis

**Figure 6:**
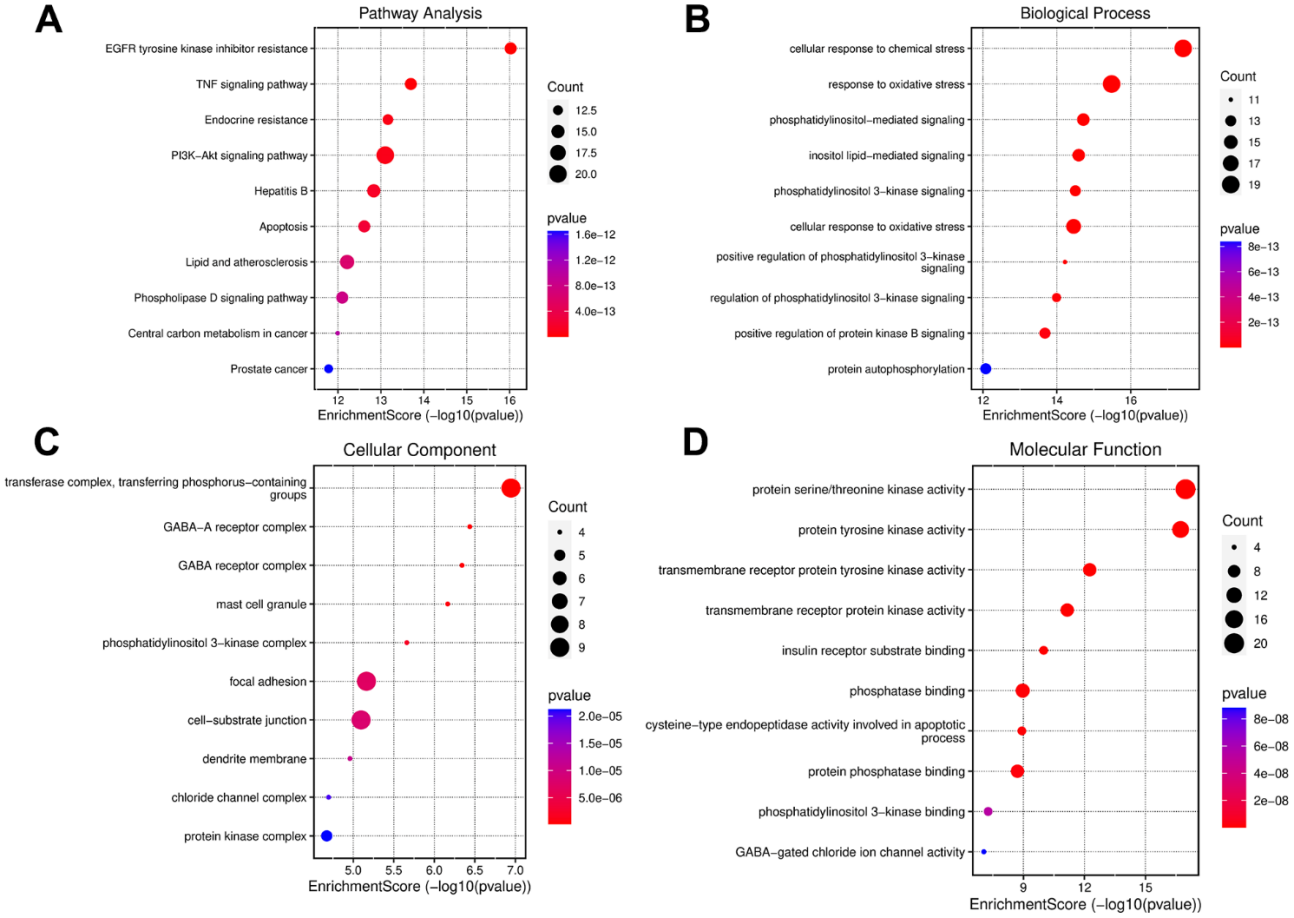
**Enrichment analysis of small molecule drug X5 with autophagy.** (**A**) KEGG enrichment analysis. (**B**–**D**) GO enrichment analysis.

Enrichment analysis of the 78 common targets was performed using the DAVID system. KEGG enrichment analysis revealed several enriched signaling pathways, and the top 10 pathways (with the smallest P-values) selected for further analysis and depicted in Figure 6A. The results revealed potential involvement of the PI3K-AKT signaling pathway, endocrine resistance, and TNF signaling pathway. Additionally, GO functional annotation yielded 298 feedback terms, of which 122 terms had a P-value < 0.01. The top 10 terms were extracted for further analysis. Our study revealed enriched biological processes (BP) related to cellular response to chemical stress, oxidative stress, phosphorylation regulation, and inositol lipid-mediated signaling (Figure 6B). Further, enriched cellular components (CC) were associated with transferase complexes, GAB-A receptor complexes, and GAB receptor complexes were identified (Figure 6C). Enriched molecular functions (MF) were related to serine/threonine kinase activity, protein tyrosine kinase activity, and transmembrane receptor protein tyrosine kinase activity were also observed, as illustrated in Figure 6D.

### X5 attenuated cell damage of H_2_O_2_-induced autophagy by activating SIRT1

**Figure 7:**
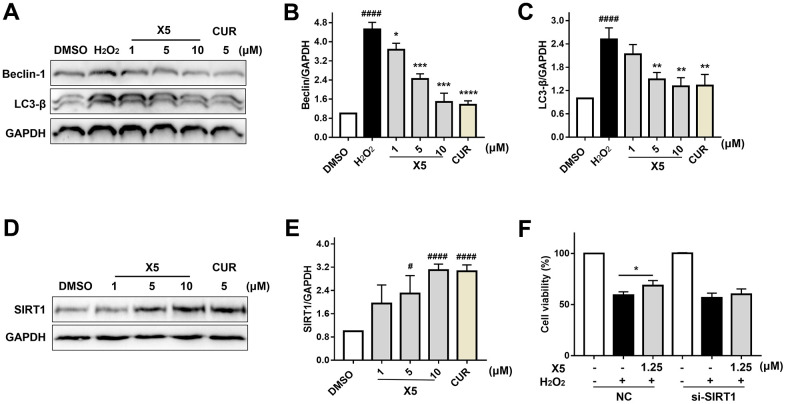
**X5 inhibited autophagy-induced injury by H_2_O_2_ in PC12 cells.** (**A**–**C**) X5 inhibited the expression of autophagy protein. PC12 cells were pre-incubated with different concentrations of X5 (1, 5 and 10 μM) and CUR (5 μM) for 18 h, then stimulated with H_2_O_2_ (800 μM) for 12 h. The protein expression of Beclin-1 and LC3-β was detected by Western blot. (**D**, **E**) X5 increased the expression of SIRT1 protein. PC12 cells were incubated with X5 (1, 5 and 10 μM) and CUR (5 μM) for 18 h, and the expression of SIRT1 protein was detected by Western blot. (**F**) SIRT1 plays an important role in H_2_O_2_-induced injury. SIRT1 was silenced in PC12 cells with siRNA, PC12 cells were incubated with X5 (1.25 μM) for 18 h, treated with H_2_O_2_ (600 μM) for 24 h, and cell viability was measured by MTT assay. The data are expressed as mean ± SD, n ≥3. ^####^p<0.0001, ^#^p<0.05 vs DMSO, ^****^p<0.0001, ^***^p<0.001, ^**^p<0.01, ^*^p<0.05 vs H_2_O_2_.

In recent years, many studies have shown that oxidative stress can induce autophagy and aggravate damage in cells [14, 20]. Furthermore, oxidative stress can induce the isolation of Beclin-1 from the antiapoptotic protein Bcl-2 to form a Beclinl-VPS34-Atg14L complex, which leads to membrane isolation and autophagic nucleation, thereby initiating autophagy to clear the damaged site [21]. Oxidative stress can promote the expression of LC3-β, a marker of autophagy which reflects the strength of autophagic activity [20]. As shown in Figure 7A–7C, H_2_O_2_ promoted the protein levels of Beclin-1 and LC3-β, but X5 inhibited their expression, suggesting that X5 could suppress oxidative stress-induced autophagy.

Many studies have shown that activation of SIRT1 can reduce oxidative stress injury and oxidative stress-induced autophagy and exert a protective effect on cells [12, 22, 23]. We found that X5 promoted SIRT1 protein expression in a concentration-dependent manner (Figure 7D–7E), but its protective effect on the cells disappeared when SIRT1 was silenced in PC12 cells through small interfering RNA (siRNA) (Figure 7F). These results indicate that SIRT1 may play an important role in the reduction of oxidative stress-induced damage by X5.

### *In vivo* protective effect of X5 in the rat MCAO model

**Figure 8:**
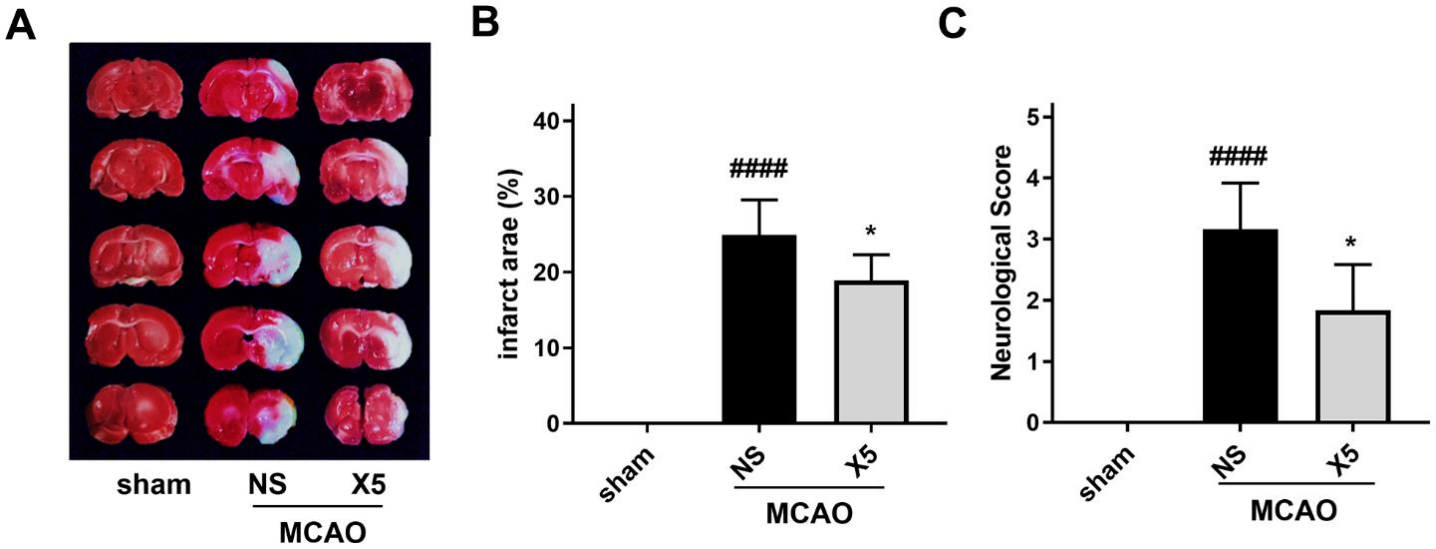
**The protective effect of compound X5 in SD rats.** (**A**) TTC staining of rat brain sections. (**B**) The statistical chart of infarct size. (**C**) Scoring of neurological function.

The middle cerebral artery occlusion (MCAO) model is commonly used to evaluate ischemic stroke *in vivo* and is widely used to screen therapeutic compounds [24, 25]. The neuroprotective effect of X5 in rats was therefore studied using the MCAO model. After 48 h of reperfusion, neurological function was evaluated by computing the neurobehavioral score, and the infarct size was measured by TTC staining. As shown in Figure 8, X5 pre-treatment not only reduced the infarct size but also improved the corresponding neurological function scores.

## DISCUSSION

Oxidative stress, a state where cellular oxidative capacities overwhelm antioxidative defenses, is involved in the pathophysiology of stroke [26]. Autophagy in the brain tissue occurs through oxidative stress-induced damage [27, 28]. The accumulation of ROS can induce autophagy and aggravate brain damage in stroke [10, 29]. The specific mechanism underlying this process may cause the excessive production of ROS leading to increased expression of autophagy substrates P62 and LC3-II/LC3-I [30], and subsequent mitochondrial damage, which activates inflammatory cytokines and pro-inflammatory cells, and ultimately leads to apoptosis [9, 27]. Therefore, reducing oxidative stress-induced excessive autophagy may be a potential therapeutic approach to alleviating CIRI.

The advancement in biotechnological techniques has empowered network pharmacology to effectively assess the relationship between various proteins. In this study, we identified 92 genes regulated by X5, 76 of which were associated with autophagy, based on network pharmacology. Through protein-protein interaction (PPI) screening, five key targets regulating autophagy by X5 were identified, namely, mTOR, AKT1, CASP3, HIF1A, and ERBB2. Of these, mTOR is a serine/threonine protein kinase, belonging to the phosphoinositide 3-kinase-related kinase (PIKK) family, that forms two distinct protein complexes known as mTOR complex 1 (mTORC1) and 2 (mTORC2). mTORC2 regulates cell survival and cytoskeletal organization, and phosphorylates AKT, leading to the activation of mTORC1 [31]. Extensive research has shown that mTORC1 plays a crucial role in autophagy, regulating different steps of the autophagic process (such as nucleation, autophagosome extension, autophagosome maturation and termination) [32–34]. Autophagy occurrence is determined by detecting autophagy-related markers such as Beclin-1, LC3-β, and Atg5 [35, 36]. Excessive ROS induces autophagy, upregulating the expression of autophagy markers Beclin-1 and LC3-β [36]. Additionally, ERBB2, a receptor tyrosine kinase, inhibits autophagy when activated, while its downregulation or inhibition promotes autophagy [37]. HIF-1α, a transcriptional regulator, promotes autophagy under hypoxic conditions [38]. ROS can activate CASP3, which directly or indirectly regulates the expression or activity of various autophagy-related proteins [39]. Our screening of mTOR, AKT1, CASP3, HIF1A, and ERBB2 through X5 suggested an association of X5 with autophagy. Subsequent experimental validation revealed that X5 can inhibit the H_2_O_2_-induced overexpression of autophagy protein Beclin-1 and that X5 has excellent antioxidant activity. Therefore, we hypothesized that X5 regulates mTOR and related molecules by inhibiting oxidative stress to inhibit autophagy, however, its specific mechanisms remain to be elucidated.

Sirtuin-1 (SIRT1) is a NAD-dependent deacetylase that is widely distributed in the cortex; it is involved in stress responses and neuropsychiatric disorders [23]. Recent studies have shown that antioxidant compounds can reduce oxidative stress-induced autophagy by activating SIRT1, which acts by reducing cell damage [40, 41]. In this study, we showed that compound X5 had a pre-protective effect in the rat MCAO model, as well as, elevated the protein level of SIRT1 and alleviated H_2_O_2_-induced cell damage, the levels of ROS, MDA and LDH, and the expression of autophagy proteins Beclin-1 and LC3-β in PC12 cells in a concentration-dependent manner. However, when SIRT1 was silenced by siRNA, compound X5 lost its protective effect in the H_2_O_2_-induced oxidative stress injury model. These results suggested that compound X5 attenuated oxidative stress-induced autophagy probably by targeting SIRT1, but the underlying mechanism remains to be explored.

In conclusion, this study leveraged the antioxidant properties of compound X5 as an investigative tool, employing a fusion of network pharmacology and experimental validation. X5 confers its antioxidant effects to inhibit oxidative stress-induced autophagy through the activation of SIRT1 protein. Furthermore, compound X5 had a pre-protective effect in the rats of MCAO, and showed a protective effect and alleviated CIRI by attenuating oxidative stress-induced autophagy.

## CONCLUSIONS

We demonstrated that the novel antioxidant compound X5 reduced oxidative stress-induced autophagy using network pharmacology and experimental verification. X5 orchestrated its effect likely by targeting SIRT1. The protective effect of X5 in the rat MCAO model underscores its potential clinical value.

## MATERIALS AND METHODS

### Cell culture

Rat adrenal pheochromocytoma cells (PC12) were purchased from the Wuhan University Cell Preservation Center. Cells were cultured in 1× Dulbecco’s modified Eagle medium (4.5 g/L D-glucose, Gibco, USA) containing 10% fetal bovine serum (Gibco, USA), and 1% penicillin-streptomycin double antibody (Gibco, USA) in an incubator (Thermo Fisher Scientific, USA) with 37°C and 5% CO_2_ after resuscitation. The cells were then digested by trypsin with 0.25% EDTA (Gibco, USA) and stored in liquid nitrogen.

### MTT assay

Cell viability was determined using the MTT assay. PC12 cells were seeded in 96-well plates (WHB Scientific, China) at a density of 5×10^3^ cells/well, treated by compounds for 18 h, and subjected to oxidative damage for 24 h with H_2_O_2_. MTT (20 μL) (3-(4,5-dimethyl-2-thiazolyl)-2,5-diphenyl-2-H-tetrazolium bromide, Thiazolyl Blue Tetrazolium Bromide, Solarbio, China) at a concentration of 5 mg/mL was added in each well and incubated under dark conditions for 4 h. Subsequently, 120 μL of dimethyl sulfoxide (DMSO) was added and the absorbance of each well was measured at 490 nm using a microorifice plate detector (Thermo Fisher Scientific, USA).

### ROS assay

PC12 cells were seeded in 6-well plates at a density of 3×10^5^ cells/well. After incubation for 24 h, PC12 cells were treated with the compounds for 18 h, and then stimulated with H_2_O_2_ for 3 h. ROS intensity was detected using a Reactive Oxygen Species Assay Kit (Beyotime, China) and cells were photographed using an inverted fluorescence microscope according to the reagent manufacturer’s method.

### MDA assay

PC12 cells were seeded in 6-well plates at a density of 3×10^5^ cells/well. After the cells adhered to the wall, they were incubated in compounds for 18 h and then in H_2_O_2_ for 6 h. MDA levels were determined using the Lipid Peroxidation MDA Assay Kit (Beyotime, China) according to the reagent manufacturer’s method.

### LDH assay

Lactate dehydrogenase (LDH) is abundant in the cytoplasm. Dead or damaged cells release LDH into the extracellular matrix. PC12 cells were seeded in 96-well plates at 5×10^3^ cells/well, and normal medium was replaced by starvation medium (without FBS). After 24 h, they were treated with compound X5 for 18 h and then subjected to oxidative damage by treatment with H_2_O_2_ for 24 h. Subsequent LDH release was measured using the LDH Release Assay Kit (Beyotime, China) according to the manufacturer’s instructions.

### Western blot analysis

PC12 cells were seeded in 6-well plates at 4.0×10^5^ cells per well, treated with X5 for 18 h, and sampled for total proteins on ice using the culture cell total protein extraction reagent (Boster Biological Technology Co. Ltd., China). Total protein was then separated by SDS-PAGE electrophoresis and the separated proteins were transferred to a polyvinylidene fluoride (PVDF) membrane (Millipore, USA). The membrane was then incubated with primary antibodies (Anti-SIRT1, Proteintech, 13161-1-AP, China, 1:1000; anti-beclin-1, Proteintech, 11306-1-AP, China, 1:1000; anti-LC3-β, Proteintech, 18725-1-AP, China, 1:1000; anti-GAPDH, Proteintech, 10494-1-AP, China, 1:1000) at 4°C for overnight. The following day, the membrane was incubated with secondary antibodies (Anti-Rabbit igg (H + L), Proteintech, SA00001-2, China, 1:2000) at room temperature for 90 min, and the immunofluorescent bands were imaged using the ChemiDoc XRS+ system (Bio-Rad, USA). Finally, the images were quantitatively analyzed using Image J (NIH, USA).

### Transfection assay

Small interfering RNA (siRNA) was obtained from the Shanghai Gemma gene with the following gene sequence: si-SIRT1 (rat); sense (5’-3’): GAGACUGCGAUGUCAUAAUTT, antisense (5’-3’): AUUAUGACAUCGCAGUCUCTT. PC12 cells were seeded in 6-well plates at 3×10^5^, and siSIRT1 was transfected using the serum-free medium and Lipofectamine™ 2000 Transfection reagent (Invitrogen, USA). Subsequently, 24 h after transfection, the medium was replaced with normal medium for conventional culture. The cells were digested with trypsin and inoculated into 96-well plates for the MTT assay.

### Experimental animals

All the adult male SD (Sprague-Dawley) rats (250–280 g) were purchased from Zhejiang Muke Biotechnology Co., Ltd. The animals were fed normally in a clean-grade animal house and provided a day-night environment at a constant temperature (25° C).

### MCAO model

SD rats were randomly divided into the sham, solvent, and drug treatment groups. All rats undergoing surgery were anesthetized with 3% sodium phenobarbital (50 mg/kg) before the procedure. Subsequently, a neck incision was made, and the common carotid artery (CCA), right external carotid artery (ECA), and internal carotid artery (ICA) were exposed. Nylon sutures were inserted through the incision from internal carotid artery until the middle cerebral artery (about 18 mm) was occluded. After 2 h of ischemia, the sutures were pulled out, blood flow and reperfusion were restored, and the incisions were sutured. Additionally, the rats were fixed in a brain stereotaxic locator and their heads were fixed stably and then injected into the lateral ventricle. X5 (0.2 mg/kg) was injected into rats using a 25 μL microinjector 2 h before MCAO, and the injector was pulled out 1 min after the injection was stopped. In the sham group, the same procedure was performed without a bolt.

### TTC staining and neurobehavioral score

Infarct size was determined in SD rats using 2,3,5- triphenyltetrazolium chloride (TTC; Sigma-Aldrich, USA). The rats were decapitated after reperfusion for 48 h, and the brains were removed and frozen in a -20°C refrigerator, followed by slices of 2 mm thickness in the brain grooves. Subsequently, brain sections were stained rose red with 2% TTC at 37°C and fixed with 4% paraformaldehyde overnight in the dark. Infarct size was quantified using Image-Pro Plus 6.0 software. Additionally, the neurobehavioral scores of SD rats were measured after reperfusion for 48 h. Neurology scores were allotted as follows: 0 for behavior similar to normal rats; 1 for difficulty extending forelimbs; 2 for circling to the right; 3 for inability to stand; and 4 for being largely unconscious and unable to move spontaneously. A higher neurobehavioral score was indicative of severe brain damage.

### Network construction

The intersection targets were imported into the STRING database (https://cn.string-db.org/), with the species limited to *Homo sapiens*. The minimum required medium confidence was set at > 0.4, to obtain the PPI network. Cytoscape was used to simplify the PPI network into the hub target network.

### GO and KEGG enrichment analysis

The intersection targets were imported into the DAVID database (https://david.ncifcrf.gov/tools.jsp) for GO and KEGG pathway enrichment analysis. The top 10 enrichment genes and pathways were then extracted from DAVID.

### Molecular docking

The crystal structure of ERK was downloaded from the RCSB Protein Data Bank (https://www.rcsb.org/), under the PDB ID 4X7K. Molecular docking studies were conducted using AutoDock Vina.

### Statistical analysis

All experiments were repeated at least three times. All data are presented as the mean ± standard deviation (SD). Statistical data were analyzed using GraphPad Prism 8.0 (GraphPad, USA) t-test or one-way ANOVA for multiple comparisons. P<0.05 was considered statistically significant.

## References

[1] Chen Y, Wright N, Guo Y, Turnbull I, Kartsonaki C, Yang L, Bian Z, Pei P, Pan D, Zhang Y, Qin H, Wang Y, Lv J, et al, and China Kadoorie Biobank Collaborative Group. Mortality and recurrent vascular events after first incident stroke: a 9-year community-based study of 0.5 million Chinese adults. Lancet Glob Health. 2020; 8:e580–90. 10.1016/S2214-109X(20)30069-3 32199124 PMC7090905

[2] Virani SS, Alonso A, Benjamin EJ, Bittencourt MS, Callaway CW, Carson AP, Chamberlain AM, Chang AR, Cheng S, Delling FN, Djousse L, Elkind MSV, Ferguson JF, et al, and American Heart Association Council on Epidemiology and Prevention Statistics Committee and Stroke Statistics Subcommittee. Heart Disease and Stroke Statistics-2020 Update: A Report From the American Heart Association. Circulation. 2020; 141:e139–596. 10.1161/CIR.0000000000000757 31992061

[3] Man S, Xian Y, Holmes DN, Matsouaka RA, Saver JL, Smith EE, Bhatt DL, Schwamm LH, Fonarow GC. Association Between Thrombolytic Door-to-Needle Time and 1-Year Mortality and Readmission in Patients With Acute Ischemic Stroke. JAMA. 2020; 323:2170–84. 10.1001/jama.2020.5697 32484532 PMC7267850

[4] Wang L, Liu Y, Zhang X, Ye Y, Xiong X, Zhang S, Gu L, Jian Z, Wang H. Endoplasmic Reticulum Stress and the Unfolded Protein Response in Cerebral Ischemia/Reperfusion Injury. Front Cell Neurosci. 2022; 16:864426. 10.3389/fncel.2022.864426 35602556 PMC9114642

[5] Chen XM, Chen HS, Xu MJ, Shen JG. Targeting reactive nitrogen species: a promising therapeutic strategy for cerebral ischemia-reperfusion injury. Acta Pharmacol Sin. 2013; 34:67–77. 10.1038/aps.2012.82 22842734 PMC4086503

[6] Vaseva AV, Marchenko ND, Ji K, Tsirka SE, Holzmann S, Moll UM. p53 opens the mitochondrial permeability transition pore to trigger necrosis. Cell. 2012; 149:1536–48. 10.1016/j.cell.2012.05.014 22726440 PMC3383624

[7] Wu X, Wei J, Yi Y, Shu G, He Z, Gong Q, Gao J. Epimedium Aqueous Extract Ameliorates Cerebral Ischemia/Reperfusion Injury through Inhibiting ROS/NLRP3-Mediated Pyroptosis. Antioxidants (Basel). 2023; 12:999. 10.3390/antiox12050999 37237865 PMC10215306

[8] Kharel P, McDonough J, Basu S. Evidence of extensive RNA oxidation in normal appearing cortex of multiple sclerosis brain. Neurochem Int. 2016; 92:43–8. 10.1016/j.neuint.2015.12.002 26706235

[9] He J, Liu J, Huang Y, Tang X, Xiao H, Hu Z. Oxidative Stress, Inflammation, and Autophagy: Potential Targets of Mesenchymal Stem Cells-Based Therapies in Ischemic Stroke. Front Neurosci. 2021; 15:641157. 10.3389/fnins.2021.641157 33716657 PMC7952613

[10] Wang P, Shao BZ, Deng Z, Chen S, Yue Z, Miao CY. Autophagy in ischemic stroke. Prog Neurobiol. 2018; 163–64:98–117. 10.1016/j.pneurobio.2018.01.001 29331396

[11] Chai P, Ni H, Zhang H, Fan X. The Evolving Functions of Autophagy in Ocular Health: A Double-edged Sword. Int J Biol Sci. 2016; 12:1332–40. 10.7150/ijbs.16245 27877085 PMC5118779

[12] Wan R, Yuan P, Guo L, Shao J, Liu X, Lai W, Kong Q, Chen L, Ge J, Xu Z, Xie J, Shen Y, Hu J, et al. Ubiquitin-like protein FAT10 suppresses SIRT1-mediated autophagy to protect against ischemic myocardial injury. J Mol Cell Cardiol. 2021; 153:1–13. 10.1016/j.yjmcc.2020.11.007 33307094

[13] Wang YT, Sansone A, Smirnov A, Stallings CL, Orvedahl A. Myeloid autophagy genes protect mice against fatal TNF- and LPS-induced cytokine storm syndromes. Autophagy. 2023; 19:1114–27. 10.1080/15548627.2022.2116675 36056542 PMC10012903

[14] Fedotova EI, Dolgacheva LP, Abramov AY, Berezhnov AV. Lactate and Pyruvate Activate Autophagy and Mitophagy that Protect Cells in Toxic Model of Parkinson’s Disease. Mol Neurobiol. 2022; 59:177–90. 10.1007/s12035-021-02583-8 34642892

[15] Zhu C, Wang X, Xu F, Bahr BA, Shibata M, Uchiyama Y, Hagberg H, Blomgren K. The influence of age on apoptotic and other mechanisms of cell death after cerebral hypoxia-ischemia. Cell Death Differ. 2005; 12:162–76. 10.1038/sj.cdd.4401545 15592434

[16] Yin Y, Sun G, Li E, Kiselyov K, Sun D. ER stress and impaired autophagy flux in neuronal degeneration and brain injury. Ageing Res Rev. 2017; 34:3–14. 10.1016/j.arr.2016.08.008 27594375 PMC5250579

[17] Hopkins AL. Network pharmacology: the next paradigm in drug discovery. Nat Chem Biol. 2008; 4:682–90. 10.1038/nchembio.118 18936753

[18] Aguayo-Orozco A, Audouze K, Brunak S, Taboureau O. In Silico Systems Pharmacology to Assess Drug’s Therapeutic and Toxic Effects. Curr Pharm Des. 2016; 22:6895–902. 10.2174/1381612822666160907093215 27604605

[19] Zhang W, Bai Y, Wang Y, Xiao W. Polypharmacology in Drug Discovery: A Review from Systems Pharmacology Perspective. Curr Pharm Des. 2016; 22:3171–81. 10.2174/1381612822666160224142812 26907941

[20] Deng M, Zhong X, Gao Z, Jiang W, Peng L, Cao Y, Zhou Z, Huang L. Dynamic changes in Beclin-1, LC3B and p62 at various time points in mice with temporary middle cerebral artery occlusion and reperfusion (tMCAO). Brain Res Bull. 2021; 173:124–31. 10.1016/j.brainresbull.2021.05.002 33974897

[21] Ma B, Cao W, Li W, Gao C, Qi Z, Zhao Y, Du J, Xue H, Peng J, Wen J, Chen H, Ning Y, Huang L, et al. Dapper1 promotes autophagy by enhancing the Beclin1-Vps34-Atg14L complex formation. Cell Res. 2014; 24:912–24. 10.1038/cr.2014.84 24980960 PMC4123296

[22] Cai Y, Feng Z, Jia Q, Guo J, Zhang P, Zhao Q, Wang YX, Liu YN, Liu WJ. Cordyceps cicadae Ameliorates Renal Hypertensive Injury and Fibrosis Through the Regulation of SIRT1-Mediated Autophagy. Front Pharmacol. 2022; 12:801094. 10.3389/fphar.2021.801094 35222012 PMC8866973

[23] Tabassum S, Misrani A, Huang HX, Zhang ZY, Li QW, Long C. Resveratrol Attenuates Chronic Unpredictable Mild Stress-Induced Alterations in the SIRT1/PGC1α/SIRT3 Pathway and Associated Mitochondrial Dysfunction in Mice. Mol Neurobiol. 2023; 60:5102–16. 10.1007/s12035-023-03395-8 37256428

[24] Longa EZ, Weinstein PR, Carlson S, Cummins R. Reversible middle cerebral artery occlusion without craniectomy in rats. Stroke. 1989; 20:84–91. 10.1161/01.str.20.1.84 2643202

[25] Gubskiy IL, Namestnikova DD, Cherkashova EA, Chekhonin VP, Baklaushev VP, Gubsky LV, Yarygin KN. MRI Guiding of the Middle Cerebral Artery Occlusion in Rats Aimed to Improve Stroke Modeling. Transl Stroke Res. 2018; 9:417–25. 10.1007/s12975-017-0590-y 29178027 PMC6061245

[26] Gibson CL, Srivastava K, Sprigg N, Bath PM, Bayraktutan U. Inhibition of Rho-kinase protects cerebral barrier from ischaemia-evoked injury through modulations of endothelial cell oxidative stress and tight junctions. J Neurochem. 2014; 129:816–26. 10.1111/jnc.12681 24528233

[27] Zhang X, Yan H, Yuan Y, Gao J, Shen Z, Cheng Y, Shen Y, Wang RR, Wang X, Hu WW, Wang G, Chen Z. Cerebral ischemia-reperfusion-induced autophagy protects against neuronal injury by mitochondrial clearance. Autophagy. 2013; 9:1321–33. 10.4161/auto.25132 23800795

[28] Samara C, Syntichaki P, Tavernarakis N. Autophagy is required for necrotic cell death in Caenorhabditis elegans. Cell Death Differ. 2008; 15:105–12. 10.1038/sj.cdd.4402231 17901876

[29] Forrester SJ, Kikuchi DS, Hernandes MS, Xu Q, Griendling KK. Reactive Oxygen Species in Metabolic and Inflammatory Signaling. Circ Res. 2018; 122:877–902. 10.1161/CIRCRESAHA.117.311401 29700084 PMC5926825

[30] Ju H, Liu C, Zhang G, Xu C, Wang H, Fan H. Neuroprotective potential of nuclear factor erythroid 2-related factor 2 (Nrf2)/antioxidant response element signaling modulator cucurbitacin I upon glucose and oxygen deprivation/reperfusion (OGD/RP). Hum Exp Toxicol. 2022; 41:9603271221104450. 10.1177/09603271221104450 35632987

[31] Saxton RA, Sabatini DM. mTOR Signaling in Growth, Metabolism, and Disease. Cell. 2017; 169:361–71. 10.1016/j.cell.2017.03.035 28388417

[32] Mendes AC, Ciccone M, Gazolla B, Bahia D. Epithelial Haven and Autophagy Breakout in Gonococci Infection. Front Cell Dev Biol. 2020; 8:439. 10.3389/fcell.2020.00439 32582714 PMC7295977

[33] Qi J, Xing Y, Liu Y, Wang MM, Wei X, Sui Z, Ding L, Zhang Y, Lu C, Fei YH, Liu N, Chen R, Wu M, et al. MCOLN1/TRPML1 finely controls oncogenic autophagy in cancer by mediating zinc influx. Autophagy. 2021; 17:4401–22. 10.1080/15548627.2021.1917132 33890549 PMC8726724

[34] Wu X, Sun R, Wang H, Yang B, Wang F, Xu H, Chen S, Zhao R, Pi J, Xu Y. Enhanced p62-NRF2 Feedback Loop due to Impaired Autophagic Flux Contributes to Arsenic-Induced Malignant Transformation of Human Keratinocytes. Oxid Med Cell Longev. 2019; 2019:1038932. 10.1155/2019/1038932 31781319 PMC6875345

[35] Khaleque MA, Kim JH, Lee HH, Kim GH, You WY, Lee WJ, Kim YY. Comparative Analysis of Autophagy and Apoptosis in Disc Degeneration: Understanding the Dynamics of Temporary-Compression-Induced Early Autophagy and Sustained-Compression-Triggered Apoptosis. Int J Mol Sci. 2024; 25:2352. 10.3390/ijms25042352 38397026 PMC10889391

[36] Shang Y, Wang Q, Li J, Liu H, Zhao Q, Huang X, Dong H, Chen W, Gui R, Nie X. Zirconia Nanoparticles Induce HeLa Cell Death Through Mitochondrial Apoptosis and Autophagy Pathways Mediated by ROS. Front Chem. 2021; 9:522708. 10.3389/fchem.2021.522708 33796503 PMC8007972

[37] Wang C, Zeng J, Li LJ, Xue M, He SL. Cdc25A inhibits autophagy-mediated ferroptosis by upregulating ErbB2 through PKM2 dephosphorylation in cervical cancer cells. Cell Death Dis. 2021; 12:1055. 10.1038/s41419-021-04342-y 34743185 PMC8572225

[38] Wei R, Song L, Miao Z, Liu K, Han G, Zhang H, Ma D, Huang J, Tian H, Xiao B, Ma C. Hydroxysafflor Yellow A Exerts Neuroprotective Effects via HIF-1α/BNIP3 Pathway to Activate Neuronal Autophagy after OGD/R. Cells. 2022; 11:3726. 10.3390/cells11233726 36496986 PMC9736542

[39] Xu YN, Cui XS, Sun SC, Lee SE, Li YH, Kwon JS, Lee SH, Hwang KC, Kim NH. Mitochondrial dysfunction influences apoptosis and autophagy in porcine parthenotes developing *in vitro*. J Reprod Dev. 2011; 57:143–50. 10.1262/jrd.10-110h 21071887

[40] Zhao Y, Shi X, Wang J, Mang J, Xu Z. Betulinic Acid Ameliorates Cerebral Injury in Middle Cerebral Artery Occlusion Rats through Regulating Autophagy. ACS Chem Neurosci. 2021; 12:2829–37. 10.1021/acschemneuro.1c00198 34296845

[41] Hsu MY, Hsiao YP, Lin YT, Chen C, Lee CM, Liao WC, Tsou SC, Lin HW, Chang YY. Quercetin Alleviates the Accumulation of Superoxide in Sodium Iodate-Induced Retinal Autophagy by Regulating Mitochondrial Reactive Oxygen Species Homeostasis through Enhanced Deacetyl-SOD2 via the Nrf2-PGC-1α-Sirt1 Pathway. Antioxidants (Basel). 2021; 10:1125. 10.3390/antiox10071125 34356358 PMC8301007

